# Supplementary material for: Economic shocks predict increases in child wasting prevalence
Source: Nat Commun. 2022 Apr 20;13:2157. doi: 10.1038/s41467-022-29755-x (PMC9021262; doi:10.1038/s41467-022-29755-x)
Supplement: Supplementary file 1 — Supplementary Information [file 41467_2022_29755_MOESM1_ESM.pdf]

Supplementary material for "Economic shocks predict increases in child wasting prevalence" by Derek D. Headey and Marie T. Ruel

CONTENTS

Supplementary Table 1: Descriptive statistics for key variables 2
Supplementary Table 2: Wasting prevalence, GNI growth and GDP growth by country and DHS round .. 3
Supplementary Table 3: Pooled sample sizes by country 7
Supplementary Fig. 1: Local polynomial estimates of moderate/severe wasting (WHZ< -2) prevalence against child age for various regions in Asia and sub-Saharan Africa (95% confidence intervals) 9
Supplementary Fig 2: Histograms of lagged annual changes in GNI and GDP per capita across 171 surveys covering 1990-2018 10
Panel A: Lagged annual changes in GNI growth 10
Panel B: Lagged annual changes in GDP growth 10
Supplementary Fig 3: Variation of the elasticity of wasting with respect to GNI growth for different specifications and sample restrictions 11
Panel A: Any wasting 11
Panel B: Moderate/severe wasting 12
Panel C: Severe wasting 13
Supplementary Fig. 4: Variation of the elasticity of wasting with respect to GDP growth for different specifications and sample restrictions 14
Panel A: Any wasting 14
Panel B: Moderate/severe wasting 15
Panel C: Severe wasting 16
Supplementary Table 4: Multivariate linear probability models of wasting risks as a function of lagged GNI growth shocks, with interactions for urban location and female status (children 0-59 months) 17
Supplementary Table 5: Multivariate linear probability models of wasting risks as a function of lagged GDP growth shocks, with interactions for urban location and female status (children 0-59 months) 18
Supplementary Fig. 5: Elasticities of children's consumption of various foods with respect to GNI growth or GDP growth (90% CIs) 19
Panel A: GNI growth 19
Panel B: GDP growth 20
Supplementary Table 6: Associations between moderate/severe wasting and morbidity symptoms, maternal underweight and child minimum dietary diversity 21

Supplementary Table 1: Descriptive statistics for key variables

Variable	N	Mean	Std. Dev.	Min	Max
Nutrition outcomes					
Any Wasting (WHZ<-1)	1,256,076	0.28	0.45	0.00	1.00
Moderate/severe wasting (WHZ<-2)	1,256,076	0.11	0.31	0.00	1.00
Severe Wasting (WHZ<-3)	1,256,076	0.04	0.20	0.00	1.00
National-level indicators					
Growth in GDP per capita (%) (UN), lag 1 year	171	2.61	6.18	-14.48	57.66
Growth in GNI per capita (%) (UN), lag 1 year	171	2.32	6.83	-28.75	52.31
Total annual rainfall (mm), lag 1 year	171	1105.93	704.74	21.53	3342.30
Average temperature (C), lag 1 year	171	22.56	5.44	3.79	29.32
Battle-related deaths per 100,000 people	171	0.47	2.14	0.00	24.65
Individual, mother, household indicators					
Household owns no DHS assets	1,256,076	0.34	0.47	0.00	1.00
Household owns some (< 5 y) assets	1,256,076	0.58	0.49	0.00	1.00
Household owns all 5 assets	1,256,076	0.07	0.25	0.00	1.00
Mother has 9 or more years of schooling	1,256,076	0.27	0.44	0.00	1.00
Household has piped water	1,256,076	0.40	0.49	0.00	1.00
Household has flush toilet	1,256,076	0.30	0.46	0.00	1.00
Child born in medical facility	1,256,076	0.60	0.49	0.00	1.00
Mother received 4 or more ANC visits	1,256,076	0.55	0.50	0.00	1.00
Child received all vaccinations	1,256,076	0.47	0.50	0.00	1.00
Teenage mother (at birth)	1,256,076	0.18	0.38	0.00	1.00
Mother has 4 or more children	1,256,076	0.39	0.49	0.00	1.00
Household is rural	1,256,076	0.67	0.47	0.00	1.00
Child is girl	1,256,076	0.49	0.50	0.00	1.00
Wasting mechanisms					
Child had diarrhea in past 2 weeks	1,230,393	0.15	0.35	0.00	1.00
Child had fever-only in past 2 weeks	1,230,393	0.09	0.28	0.00	1.00
Low maternal BMI (<18.5)	884,436	0.12	0.32	0.00	1.00
Child meets minimum dietary diversity (MDD)	323,014	0.32	0.47	0.00	1.00
Starchy staples in past 24 hours	323,014	0.72	0.45	0.00	1.00
Legumes/nuts in past 24 hours	323,014	0.24	0.42	0.00	1.00
Dairy in past 24 hours	323,014	0.42	0.49	0.00	1.00
Flesh foods in past 24 hours	323,014	0.34	0.48	0.00	1.00
Eggs in past 24 hours	323,014	0.20	0.40	0.00	1.00
Vitamin A-rich fruits/vegetables in past 24 hours	323,014	0.43	0.49	0.00	1.00
Other fruits/vegetables in past 24 hours	323,014	0.27	0.44	0.00	1.00

N = 1,256,076 children for the main DHS variables of interest, *N* = 171 country-level growth shocks and other shocks, and *N* = 1,230,393 for child diarrhea and fever-only in the past 2 weeks, *N* = 884,436 for Low maternal BMI, and *N* = 323, 014 for child dietary data. GDP=Gross Domestic Product and GNI=Gross National Income. WHZ=Weight for height Z score. BMI equals Body Mass Index. ANC equals antenatal care.

Supplementary Table 2: Wasting prevalence, GNI growth and GDP growth by country and DHS round

Country	Survey year(s)	Any wasting (WHZ<-1)	Moderate/severe wasting (WHZ<-2)	Severe wasting (WHZ<-3)	GNI growth (lag 1 year)	GDP growth (lag 1 year)
Albania	2008-2009	9.9%	17.9%	5.8%	6.4%	8.0%
Albania	2017-2018	2.2%	7.6%	0.8%	3.6%	3.5%
Armenia	2000-2000	2.3%	7.2%	0.7%	3.8%	3.9%
Armenia	2005-2005	5.3%	12.6%	2.7%	15.7%	11.1%
Armenia	2010-2010	4.3%	11.6%	2.0%	-15.3%	-13.6%
Armenia	2015-2016	5.2%	11.1%	2.1%	2.2%	2.9%
Bangladesh	1996-1997	21.3%	48.4%	7.8%	2.5%	2.6%
Bangladesh	1999-2000	12.7%	44.1%	2.8%	3.0%	2.9%
Bangladesh	2004-2004	14.7%	46.5%	3.7%	4.4%	3.4%
Bangladesh	2007-2007	17.3%	52.0%	3.5%	5.7%	5.2%
Bangladesh	2011-2011	15.9%	48.4%	4.3%	4.6%	4.4%
Bangladesh	2014-2014	15.8%	44.8%	4.3%	4.4%	4.8%
Benin	2001-2001	10.2%	26.8%	3.9%	2.7%	2.7%
Benin	2006-2006	9.1%	23.0%	3.7%	-0.8%	-1.2%
Benin	2011-2012	17.6%	29.9%	10.1%	0.4%	0.1%
Benin	2017-2018	5.2%	23.3%	1.2%	2.6%	2.6%
Bolivia	1998-1998	2.0%	6.5%	0.9%	3.5%	2.9%
Bolivia	2003-2003	2.2%	6.8%	1.0%	0.4%	0.6%
Bolivia	2008-2008	1.7%	6.2%	0.7%	5.6%	2.8%
Burkina Faso	1993-1993	14.9%	36.3%	5.9%	-1.0%	-0.9%
Burkina Faso	1998-1999	15.8%	37.4%	6.0%	3.9%	4.1%
Burkina Faso	2003-2003	24.2%	43.4%	12.7%	1.6%	1.4%
Burkina Faso	2010-2010	16.0%	37.6%	6.5%	-1.0%	-0.1%
Burundi	2010-2010	6.3%	23.0%	1.8%	-0.1%	0.5%
Burundi	2016-2017	5.1%	23.4%	0.9%	-4.1%	-2.1%
Cambodia	2000-2000	17.8%	41.7%	8.5%	8.0%	9.2%
Cambodia	2005-2005	8.7%	35.1%	1.7%	9.9%	9.6%
Cambodia	2010-2010	12.1%	36.8%	3.9%	-1.5%	-1.3%
Cambodia	2014-2014	10.2%	36.0%	3.3%	6.7%	5.7%
Cameroon	2004-2004	6.2%	16.8%	2.4%	4.6%	1.8%
Cameroon	2011-2011	5.8%	17.7%	2.0%	3.7%	0.6%
Chad	2004-2004	18.6%	40.5%	8.4%	-5.6%	10.0%
Chad	2014-2015	15.3%	38.2%	5.3%	-3.3%	-2.9%
Colombia	1995-1995	1.8%	10.2%	0.6%	5.9%	3.2%
Colombia	2000-2000	1.3%	7.5%	0.5%	-5.8%	-5.8%
Colombia	2005-2005	2.2%	11.8%	0.7%	2.8%	3.5%
Colombia	2010-2010	1.2%	8.6%	0.3%	0.6%	0.3%
Congo Rep.	2005-2005	8.5%	21.5%	4.0%	-7.3%	0.5%
Congo Rep.	2011-2012	5.7%	22.1%	1.9%	12.4%	9.5%
Congo DRC	2007-2007	11.1%	27.0%	5.7%	-0.8%	2.0%
Congo DRC	2013-2014	8.8%	25.0%	3.6%	3.1%	3.9%
Cote d'Ivoire	1998-1999	6.7%	22.4%	2.3%	4.1%	2.2%
Cote d'Ivoire	2011-2012	7.4%	23.1%	2.1%	-6.1%	-6.0%
Dominican Rep.	1996-1996	2.3%	11.5%	0.7%	5.0%	3.7%
Dominican Rep.	2002-2002	2.3%	10.5%	0.9%	0.3%	0.3%
Dominican Rep.	2007-2007	2.2%	10.2%	0.8%	9.7%	9.2%
Dominican Rep.	2013-2013	2.6%	12.6%	0.8%	1.4%	1.5%
Egypt	1992-1992	4.2%	9.9%	2.0%	4.6%	3.0%
Egypt	1995-1995	6.4%	14.2%	3.0%	2.9%	1.9%
Egypt	2000-2000	3.2%	8.2%	1.6%	4.0%	4.0%

Egypt	2003-2003	5.1%	16.4%	1.9%	0.5%	1.3%
Egypt	2005-2005	5.7%	13.2%	2.7%	2.1%	2.2%
Egypt	2008-2008	8.3%	16.0%	4.4%	7.7%	5.2%
Egypt	2014-2014	13.0%	23.1%	7.6%	-0.8%	-0.1%
Ethiopia	2000-2000	14.3%	38.6%	4.9%	-10.4%	-10.4%
Ethiopia	2005-2005	13.7%	34.6%	5.7%	10.4%	10.0%
Ethiopia	2011-2011	12.2%	36.0%	3.6%	-1.3%	-1.4%
Ethiopia	2016-2016	12.4%	35.6%	3.7%	7.2%	7.4%
Gabon	2000-2000	4.5%	15.1%	1.7%	-28.5%	-12.7%
Gabon	2012-2012	4.6%	15.8%	1.8%	10.6%	3.2%
Ghana	1998-1998	10.3%	31.0%	2.9%	1.8%	1.9%
Ghana	2003-2003	9.6%	26.2%	3.4%	2.2%	2.3%
Ghana	2008-2008	10.1%	28.9%	3.6%	1.8%	1.7%
Ghana	2014-2014	5.1%	24.1%	1.1%	8.1%	4.8%
Guatemala	1995-1995	4.3%	13.2%	1.8%	1.8%	1.6%
Guatemala	1998-1999	3.0%	10.1%	1.4%	2.9%	2.4%
Guatemala	2014-2015	0.7%	7.1%	0.1%	1.8%	1.9%
Guinea	1999-1999	10.5%	28.0%	4.8%	1.5%	1.9%
Guinea	2005-2005	11.5%	27.7%	4.7%	-0.1%	0.3%
Guinea	2012-2012	11.4%	29.1%	5.3%	5.0%	3.3%
Guinea	2018-2018	9.4%	25.7%	4.1%	10.2%	10.3%
Haiti	2000-2000	6.2%	20.4%	2.1%	0.7%	0.9%
Haiti	2005-2006	9.6%	23.4%	3.7%	-1.4%	-2.0%
Haiti	2012-2012	5.4%	21.0%	1.7%	4.1%	3.9%
Haiti	2016-2017	3.8%	16.4%	1.0%	0.2%	0.1%
Honduras	2005-2006	1.5%	8.5%	0.4%	3.8%	3.6%
Honduras	2011-2012	1.5%	9.2%	0.3%	0.1%	1.8%
India	2005-2006	19.3%	46.4%	7.4%	6.2%	6.2%
India	2015-2016	21.2%	47.9%	8.8%	6.5%	6.5%
Jordan	1990-1990	4.5%	12.9%	1.8%	-18.2%	-14.5%
Jordan	1997-1997	2.4%	11.7%	0.6%	-0.8%	-1.0%
Jordan	2002-2002	2.5%	12.0%	0.8%	4.0%	3.4%
Jordan	2012-2012	2.4%	10.7%	0.9%	-2.7%	-2.6%
Kazakhstan	1995-1995	6.5%	16.4%	2.0%	-11.9%	-11.7%
Kazakhstan	1999-1999	3.0%	12.3%	1.2%	-0.4%	-0.5%
Kenya	1993-1993	7.5%	22.1%	2.9%	-3.1%	-3.9%
Kenya	1998-1998	8.8%	23.6%	4.1%	-2.5%	-2.5%
Kenya	2003-2003	7.4%	21.3%	3.3%	-2.3%	-2.1%
Kenya	2008-2009	8.9%	24.0%	3.4%	0.5%	0.4%
Kenya	2014-2014	5.8%	21.6%	1.6%	2.7%	3.2%
Lesotho	2004-2004	6.1%	16.0%	3.4%	3.8%	5.1%
Lesotho	2009-2010	4.9%	16.3%	2.2%	3.8%	6.2%
Lesotho	2014-2014	3.9%	12.5%	1.4%	-0.2%	1.2%
Liberia	2007-2007	8.4%	21.7%	3.8%	14.4%	9.2%
Liberia	2013-2013	6.8%	21.6%	2.6%	-0.1%	8.1%
Madagascar	1992-1992	6.3%	25.2%	1.3%	-11.2%	-9.0%
Madagascar	1997-1997	10.1%	31.0%	3.2%	0.1%	-1.0%
Madagascar	2003-2004	15.5%	37.1%	6.3%	-0.7%	-0.7%
Malawi	1992-1992	5.9%	17.1%	2.1%	7.4%	5.6%
Malawi	2000-2000	6.9%	17.8%	3.0%	-0.9%	-1.6%
Malawi	2004-2004	7.0%	16.3%	3.6%	2.3%	3.1%
Malawi	2010-2010	4.5%	13.8%	1.8%	6.1%	5.3%
Malawi	2015-2016	3.4%	14.3%	0.9%	2.4%	2.4%
Maldives	2009-2009	12.1%	37.2%	3.5%	6.9%	6.8%
Maldives	2016-2017	10.1%	36.2%	2.6%	1.1%	1.2%
Mali	2006-2006	17.4%	37.3%	7.8%	6.9%	6.9%

Mali	2012-2013	13.8%	32.7%	6.2%	5.9%	5.8%
Mali	2018-2018	9.9%	32.8%	3.2%	4.2%	3.7%
Morocco	1992-1992	2.8%	8.7%	1.3%	4.6%	5.0%
Morocco	2003-2004	11.9%	22.1%	6.1%	3.2%	3.0%
Mozambique	2003-2003	5.4%	15.8%	2.4%	1.1%	5.6%
Mozambique	2011-2011	5.7%	16.4%	2.1%	10.9%	3.8%
Namibia	1992-1992	9.5%	28.0%	3.2%	6.0%	5.0%
Namibia	2000-2000	10.7%	31.8%	3.6%	-1.8%	1.5%
Namibia	2006-2007	8.4%	27.0%	2.7%	4.1%	4.2%
Namibia	2013-2013	8.9%	27.4%	3.4%	1.8%	3.2%
Nepal	2001-2001	10.8%	38.6%	2.7%	4.5%	4.2%
Nepal	2006-2006	12.7%	42.1%	3.0%	2.8%	2.2%
Nepal	2011-2011	10.9%	37.5%	2.9%	3.9%	4.3%
Nepal	2016-2016	9.8%	36.8%	2.0%	2.6%	2.7%
Nicaragua	1998-1998	3.3%	10.8%	1.6%	4.8%	2.6%
Nicaragua	2001-2001	2.5%	9.3%	1.1%	2.6%	2.5%
Niger	2006-2006	13.3%	35.0%	5.1%	3.7%	3.5%
Niger	2012-2012	19.5%	44.3%	8.4%	-1.6%	-1.6%
Nigeria	1990-1990	10.9%	27.9%	4.2%	0.2%	-0.7%
Nigeria	2003-2003	12.4%	29.1%	5.8%	14.5%	12.5%
Nigeria	2008-2008	16.9%	30.2%	10.1%	-1.9%	3.8%
Nigeria	2013-2013	17.9%	36.3%	9.2%	1.6%	1.5%
Pakistan	1990-1991	11.1%	28.2%	4.1%	1.9%	1.5%
Pakistan	2012-2013	11.0%	29.1%	4.5%	1.4%	0.8%
Pakistan	2017-2018	9.5%	25.8%	4.2%	3.0%	3.5%
Peru	1991-1992	2.3%	7.9%	0.9%	-5.3%	-3.8%
Peru	1996-1996	2.3%	6.9%	1.1%	5.1%	5.4%
Peru	2000-2000	1.8%	6.4%	0.7%	-0.2%	-0.1%
Peru	2003-2008	1.1%	4.8%	0.3%	6.2%	6.6%
Peru	2009-2009	0.8%	5.0%	0.3%	9.2%	8.3%
Peru	2010-2010	0.8%	5.7%	0.2%	1.0%	0.3%
Peru	2011-2011	0.6%	5.2%	0.1%	6.2%	7.5%
Peru	2012-2012	0.7%	5.7%	0.2%	5.4%	5.5%
Rwanda	1992-1992	5.1%	17.7%	2.0%	-1.0%	-0.9%
Rwanda	2000-2000	9.1%	21.6%	4.1%	-0.9%	-1.2%
Rwanda	2005-2005	5.0%	14.6%	1.7%	6.1%	5.9%
Rwanda	2010-2010	3.0%	10.9%	1.0%	4.0%	4.0%
Rwanda	2014-2015	2.4%	9.2%	0.9%	3.5%	4.0%
Senegal	1992-1993	9.8%	28.4%	3.4%	-1.2%	-1.6%
Senegal	2005-2005	9.4%	30.9%	2.6%	3.5%	3.3%
Senegal	2010-2011	10.2%	34.0%	3.0%	0.8%	0.7%
Senegal	2012-2013	10.4%	36.5%	2.8%	-1.3%	0.4%
Senegal	2014-2014	7.0%	30.5%	1.1%	5.0%	1.0%
Senegal	2017-2017	9.7%	36.8%	1.7%	3.5%	3.4%
Sierra Leone	2008-2008	11.7%	25.9%	5.9%	2.0%	5.2%
Sierra Leone	2013-2013	10.5%	21.9%	5.4%	17.9%	12.6%
Tajikistan	2012-2012	10.6%	25.2%	4.8%	-4.2%	0.1%
Tajikistan	2017-2017	6.7%	19.3%	2.4%	-3.3%	4.4%
Tanzania	1991-1992	8.7%	23.1%	3.4%	3.7%	3.2%
Tanzania	1996-1996	9.2%	25.4%	3.6%	1.2%	0.6%
Tanzania	1999-1999	5.9%	21.7%	2.4%	2.2%	2.0%
Tanzania	2004-2005	4.5%	18.2%	1.5%	3.8%	4.2%
Tanzania	2010-2010	6.8%	20.8%	2.3%	2.6%	2.4%
Tanzania	2015-2016	5.1%	19.5%	1.5%	3.7%	3.4%
Turkey	1993-1993	3.6%	11.5%	1.0%	4.5%	4.3%
Turkey	1998-1998	3.7%	12.2%	1.4%	6.4%	5.8%

Turkey	2003-2003	1.1%	4.9%	0.3%	4.1%	4.3%
Uganda	2000-2001	5.1%	17.0%	1.7%	1.7%	2.5%
Uganda	2006-2006	7.1%	20.1%	2.5%	6.3%	6.6%
Uganda	2011-2011	5.9%	20.4%	2.0%	4.5%	4.8%
Uganda	2016-2016	4.1%	15.8%	1.6%	2.7%	2.1%
Yemen	1991-1992	15.4%	35.5%	6.6%	12.8%	13.1%
Yemen	2013-2013	17.0%	43.8%	6.3%	3.1%	-0.6%
Zambia	1992-1992	6.7%	19.1%	2.6%	-7.4%	-4.5%
Zambia	1996-1996	5.7%	17.3%	2.0%	0.5%	0.4%
Zambia	2001-2002	6.5%	19.9%	2.5%	2.7%	2.3%
Zambia	2007-2007	6.6%	17.8%	3.1%	3.3%	5.1%
Zambia	2013-2014	6.9%	19.2%	3.0%	10.3%	3.3%
Zimbabwe	1999-1999	9.3%	21.5%	5.2%	-2.0%	-0.3%
Zimbabwe	2005-2006	7.6%	19.4%	3.3%	-4.5%	-4.1%
Zimbabwe	2010-2011	3.8%	15.1%	1.3%	36.9%	38.9%
Zimbabwe	2015-2015	3.8%	13.6%	1.5%	1.0%	0.6%

Wasting estimates are from the authors’ estimates from DHS data, while lagged GNI and GDP growth are sourced from the UN. GDP=Gross Domestic Product and GNI=Gross National Income. WHZ=Weight for height Z score.

Supplementary Table 3: Pooled sample sizes by country

Country	First year	Last Year	Total Observations	Share of total < 5 years observations in this DHS dataset	Share of total < 5 years population in DHS countries
Albania	2008	2018	3780	0.30%	0.05%
Armenia	2000	2016	5578	0.44%	0.06%
Bangladesh	1996	2014	33293	2.65%	5.03%
Benin	2001	2018	38118	3.03%	0.43%
Bolivia	1998	2008	22499	1.79%	0.36%
Burkina Faso	1993	2010	23946	1.91%	0.74%
Burundi	2010	2017	8929	0.71%	0.49%
Cambodia	2000	2014	15104	1.20%	0.51%
Cameroon	2004	2011	8141	0.65%	1.00%
Chad	2004	2015	14268	1.14%	0.75%
Colombia	1995	2010	30359	2.42%	1.26%
Congo Rep.	2005	2012	7943	0.63%	0.21%
Congo DRC	2007	2014	11223	0.89%	3.98%
Cote d'Ivoire	1998	2012	4691	0.37%	0.90%
Dominican Rep.	1996	2013	24875	1.98%	0.32%
Egypt	1992	2014	70457	5.61%	2.98%
Ethiopia	2000	2016	30686	2.44%	4.24%
Gabon	2000	2012	6668	0.53%	0.06%
Ghana	1998	2014	10785	0.86%	1.03%
Guatemala	1995	2015	24092	1.92%	0.59%
Guinea	1999	2018	13282	1.06%	0.51%
Haiti	2000	2017	17239	1.37%	0.38%
Honduras	2005	2012	18283	1.46%	0.31%
India	2005	2016	268786	21.40%	38.14%
Jordan	1990	2012	23443	1.87%	0.24%
Kazakhstan	1995	1999	1213	0.10%	0.39%
Kenya	1993	2014	35322	2.81%	1.96%
Lesotho	2004	2014	4206	0.33%	0.08%
Liberia	2007	2013	6762	0.54%	0.19%
Madagascar	1992	2004	11554	0.92%	0.87%
Malawi	1992	2016	29972	2.39%	0.73%
Maldives	2009	2017	4006	0.32%	0.01%
Mali	2006	2018	23552	1.88%	0.78%
Morocco	1992	2004	10053	0.80%	1.00%
Mozambique	2003	2011	16879	1.34%	1.20%
Namibia	1992	2013	9835	0.78%	0.08%
Nepal	2001	2016	15800	1.26%	1.04%
Nicaragua	1998	2001	12613	1.00%	0.21%
Niger	2006	2012	8603	0.68%	0.90%

Nigeria	1990	2013	54032	4.30%	8.44%
Pakistan	1990	2018	10997	0.88%	6.48%
Peru	1991	2012	70667	5.63%	0.92%
Rwanda	1992	2015	21280	1.69%	0.46%
Senegal	1992	2017	33166	2.64%	0.67%
Sierra Leone	2008	2013	5595	0.45%	0.35%
Tajikistan	2012	2017	10392	0.83%	0.31%
Tanzania	1991	2016	36573	2.91%	2.18%
Turkey	1993	2003	9846	0.78%	2.02%
Uganda	2000	2016	13529	1.08%	1.72%
Yemen	1991	2013	15971	1.27%	1.11%
Zambia	1992	2014	31990	2.55%	0.67%
Zimbabwe	1999	2015	15200	1.21%	0.66%
Total	1990	2018	1256076	100.00%	100.00%

Wasting estimates are from the authors' estimates from DHS data, while lagged GNI and GDP growth are sourced from the UN.

Supplementary Fig. 1: Local polynomial estimates of moderate/severe wasting (WHZ< -2) prevalence against child age for various regions in Asia and sub-Saharan Africa

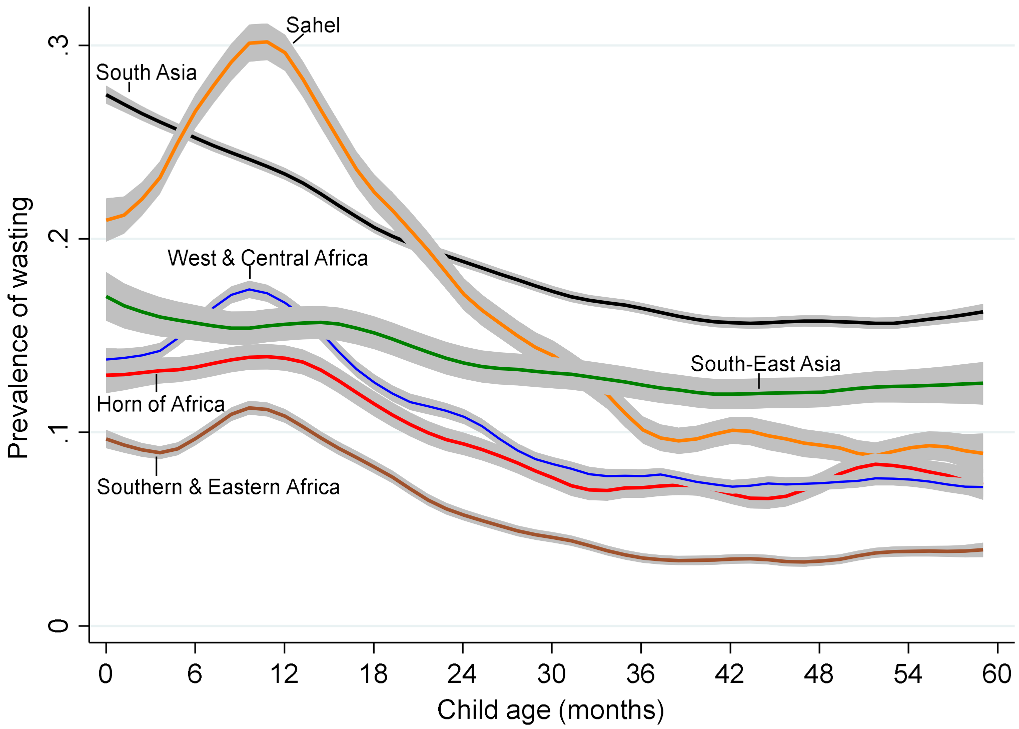

Data are presented as predicted values +/- 95% confidence interval estimates derived from local polynomial regressions of wasting (WHZ< -2) status against child age using the *lpolyci* command in STATA™. Sample sizes are as follows. South Asia: N = 402,990. South-East Asia: N = 27,614. Sahel = 80,717. West and Central Africa: N = 248,204. Horn of Africa: N = 68,608; Southern and Eastern Africa: N = 228,818. WHZ=Weight for height Z score.

Supplementary Fig 2: Histograms of lagged annual changes in GNI and GDP per capita across 171 surveys covering 1990-2018

Panel A: Lagged annual changes in GNI growth

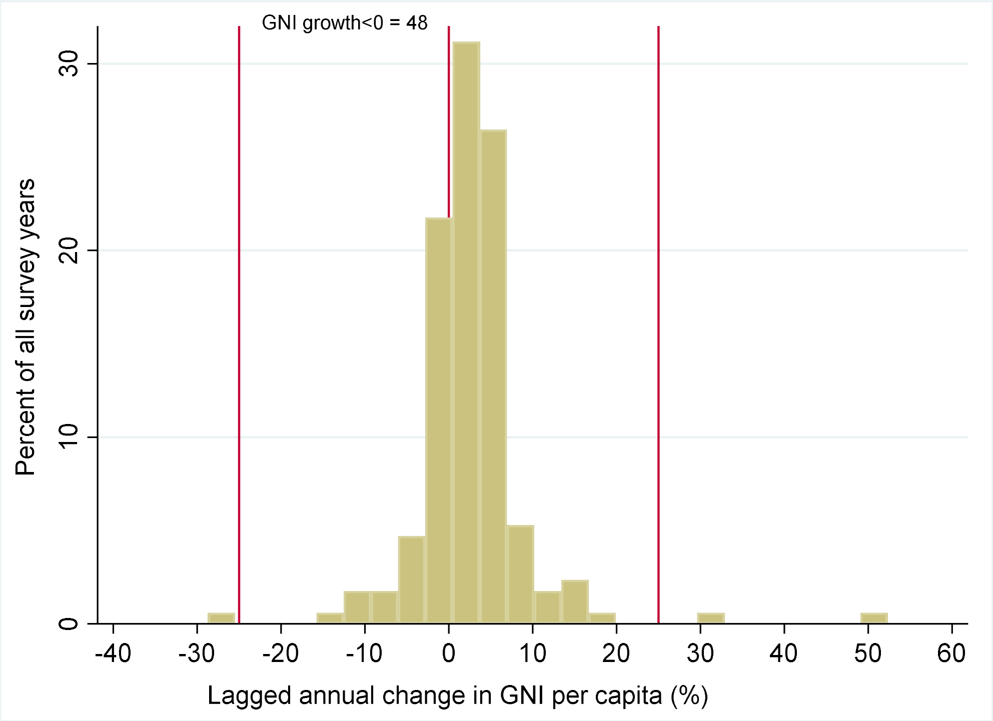

Panel B: Lagged annual changes in GDP growth

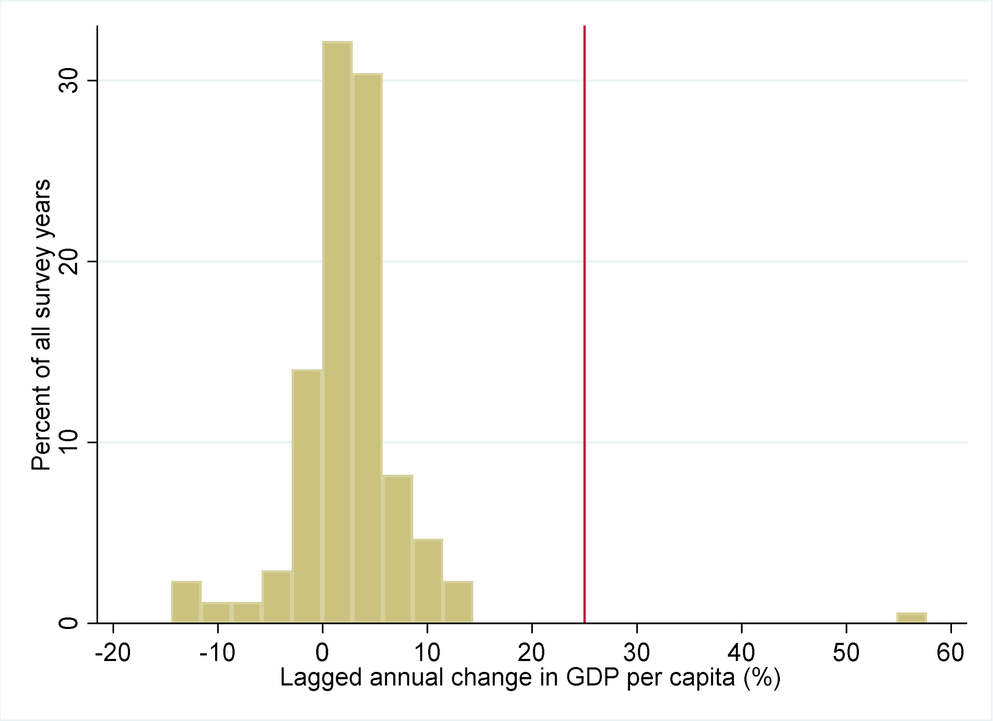

N = 171 countries. Authors' estimates of the distribution of UN data on changes in GNI and GDP per capita for the DHS survey years. Red lines show thresholds for extreme values used to omit potential outliers. GDP=Gross Domestic Product and GNI=Gross National Income.

Supplementary Fig 3: Variation of the elasticity of wasting with respect to GNI growth for different specifications and sample restrictions

Panel A: Any wasting (WHZ< -1)

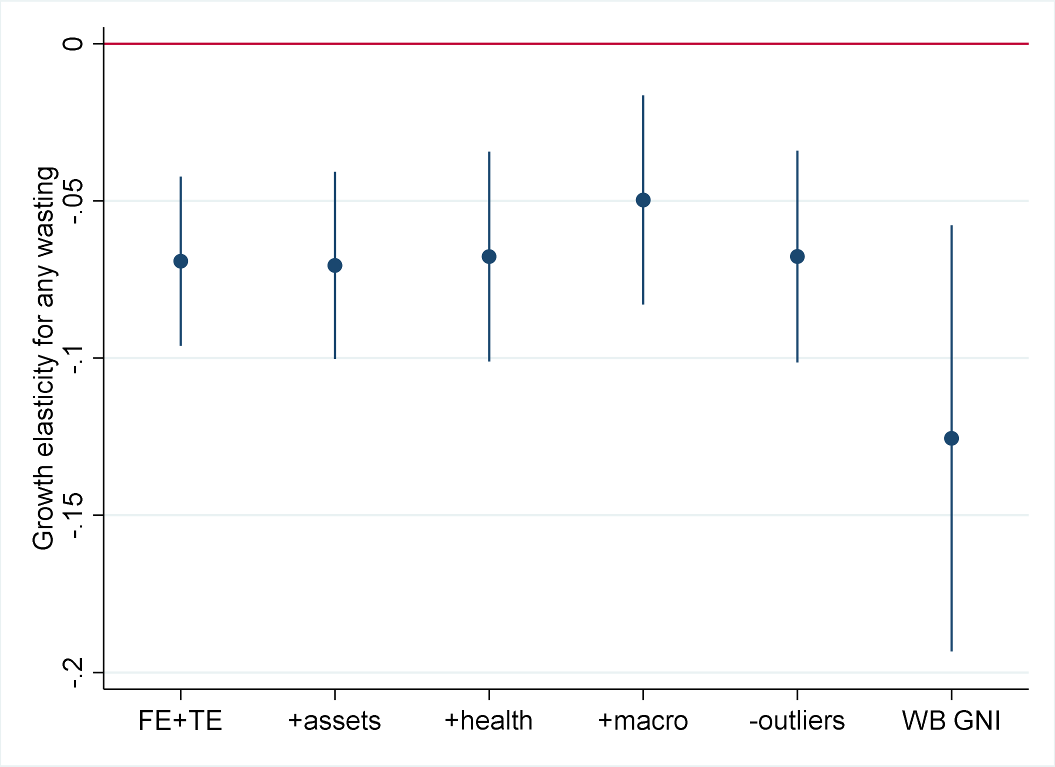

Panel B: Moderate/severe wasting (WHZ< -2)

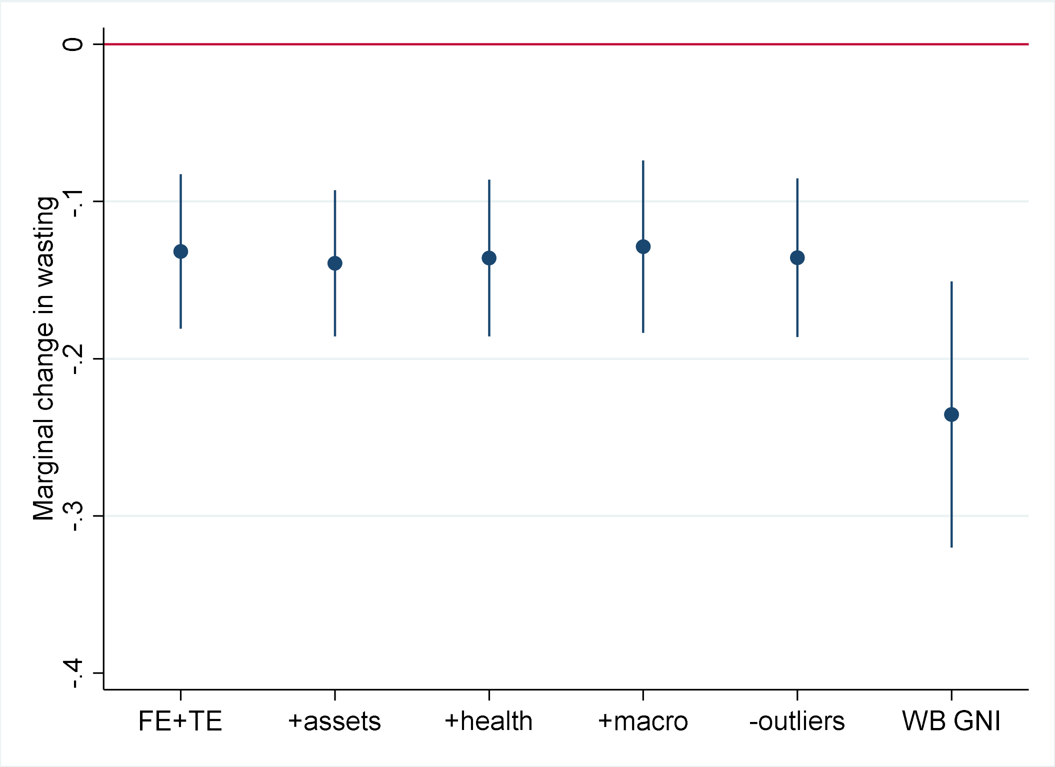

Panel C: Severe wasting (WHZ< -3)

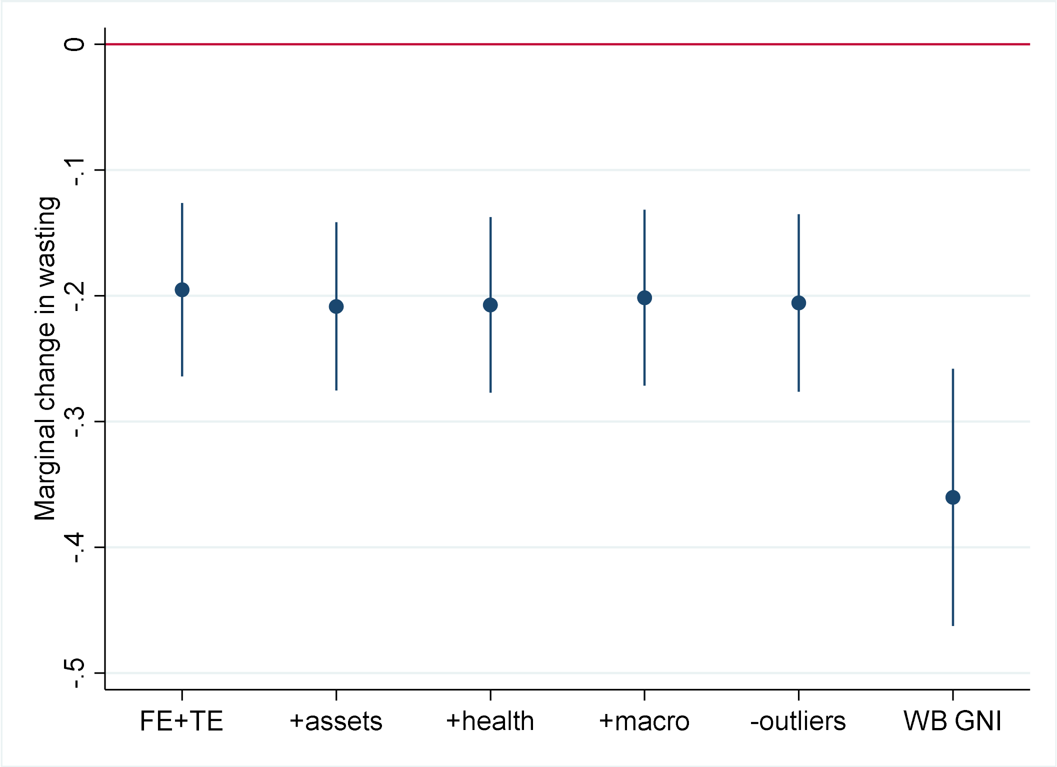

Data are presented are linear probability coefficient point estimates for lagged GNI growth with 95% confidence interval derived from the regression model outlined in equation (1). The different model specifications (and samples sizes) are specified sequentially as follows: FE+TE = fixed effects and time effects only (*N* = 1,256,076); "+ assets" adds household assets, education, toilet and water ownership to the FE+TE model (*N* = 1,256,076); "+health" adds antenatal care, medical facility birth and all vaccinations to the "+assets model" (*N* = 1,256,076); "+macro" adds controls for lagged national rainfall, temperature and battle deaths (*N* = 1,256,076); "-large values" remove GNI growth values greater than 25% in absolute magnitude (*N = 1,259,097*); "WB GNI" replaces the UN GNI measure with the World Bank GNI measure (*N* = 1,136,206). GDP=Gross Domestic Product and GNI=Gross National Income. WHZ=Weight for height Z score. WB=World Bank.

Supplementary Fig. 4: Variation of the elasticity of wasting with respect to GDP growth for different specifications and sample restrictions

Panel A: Any wasting (WHZ< -1)

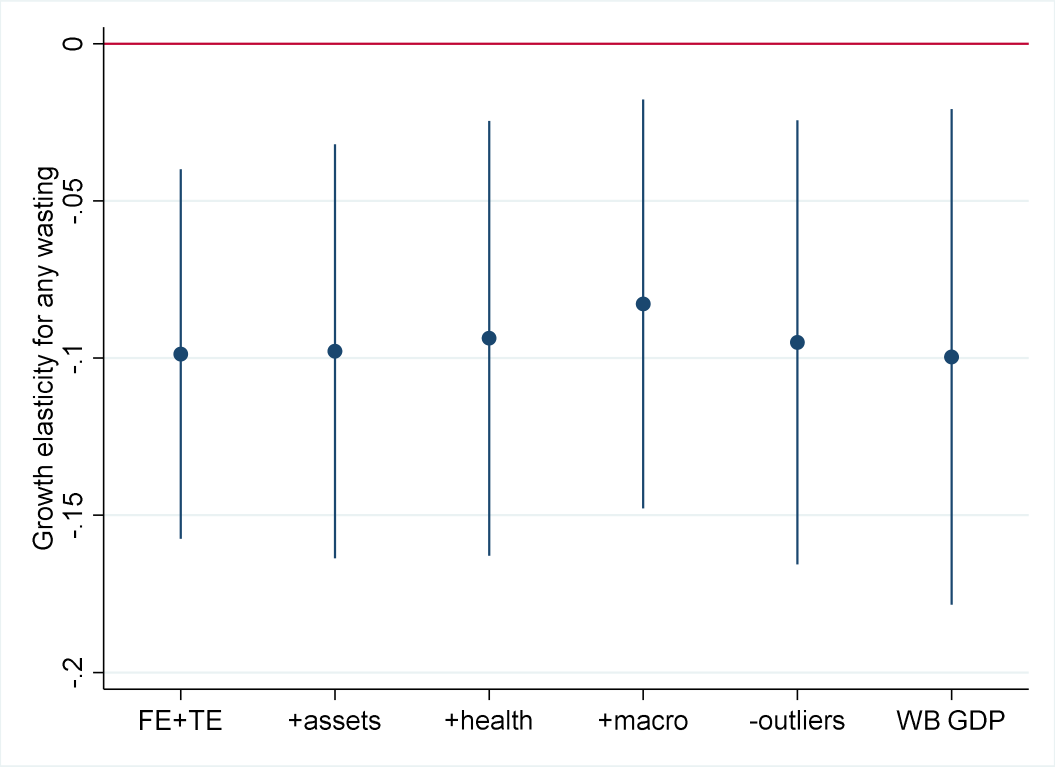

Panel B: Moderate/severe wasting (WHZ< -2)

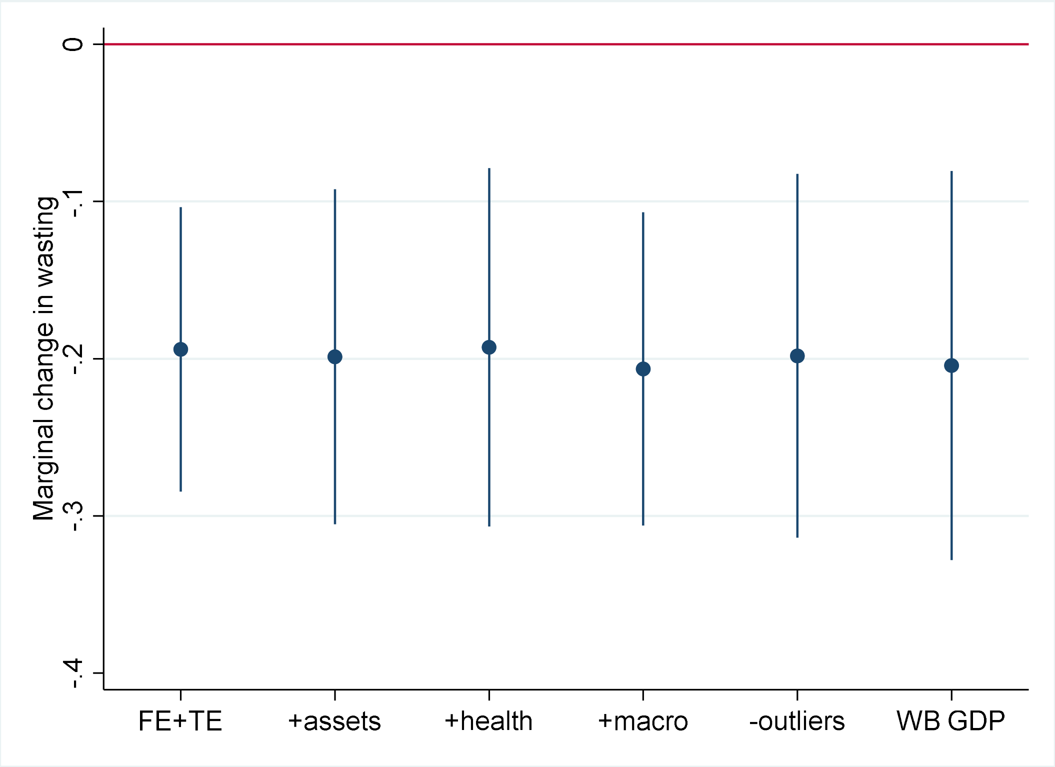

Panel C: Severe wasting (WHZ< -3)

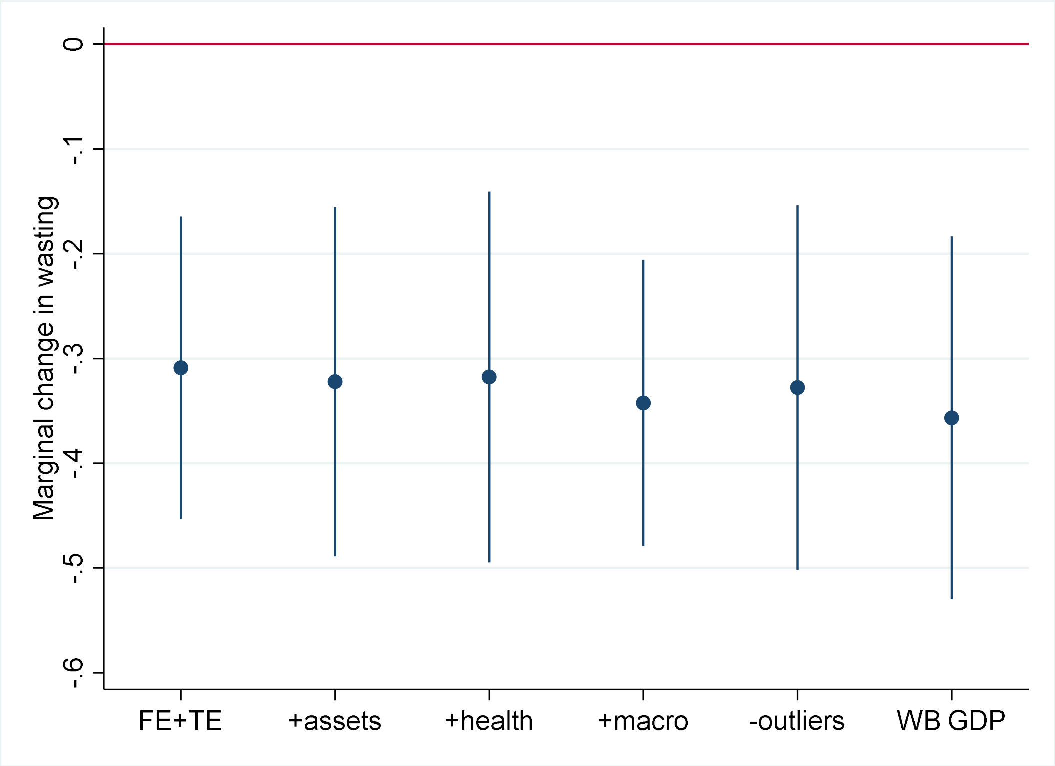

Data are presented as linear probability coefficient point estimates for lagged GDP growth with 95% confidence interval derived from least squares regressions as outlined in equation (1). The different model specifications (and samples sizes) are specified sequentially as follows: FE+TE = fixed effects and time effects only (*N* = 1,256,076); "+ assets" adds household assets, education, toilet and water ownership to the FE+TE model (*N* = 1,256,076); "+health" adds antenatal care, medical facility birth and all vaccinations to the "+assets model" (*N* = 1,256,076); "+macro" adds controls for lagged national rainfall, temperature and battle deaths (*N* = 1,256,076); "-large values" remove GNI growth values greater than 25% in absolute magnitude (*N = 1,259,097*); "WB GDP" replaces the UN GDP measure with the World Bank GDP measure (*N* = 1,136,206). GDP=Gross Domestic Product and GNI=Gross National Income. WHZ=Weight for height Z score. WB = World Bank.

Supplementary Table 4: Multivariate linear probability models of wasting risks as a function of lagged GNI growth shocks, with interactions for urban location and female status (children 0-59 months)

	Any wasting (WHZ < -1)	Moderate/severe wasting (WHZ < -2)	Severe wasting (WHZ < -3)
	(1) N=1,256,076	(2) N=1,256,076	(3) N=1,256,076
GNI growth elasticity (*w*.g^n)[a]	-0.060*** (-0.083, -0.037)	-0.148*** (-0.189, -0.106)	-0.223*** (-0.300, -0.147)
GNI growth elasticity (*w*.g^n)*urban dummy	-0.047** (-0.087, -0.006)	-0.076 (-0.187, 0.035)	0.004 (-0.103, 0.111)
R-squared	0.432	0.200	0.085
	(4) N=1,256,076	(5) N=1,256,076	(6) N=1,256,076
GNI growth elasticity (*w*.g^n)[b]	-0.079*** (-0.104, -0.054)	-0.132*** (-0.180, -0.084)	-0.197*** (-0.277, -0.118)
GNI growth elasticity (*w*.g^n)*girl dummy	0.016 (-0.012, 0.044)	-0.026 (-0.077, 0.026)	-0.050 (-0.135, 0.035)
R-squared	0.432	0.200	0.085
DHS child, mother, household effects?[c]	Yes	Yes	Yes
Country fixed effects?	Yes	Yes	Yes
Region-specific temporal effects?[d]	Yes	Yes	Yes

Notes: *N* = 1,256,076 children in all regressions. Results are linear probability coefficient point estimates with 95% confidence intervals based on standard errors clustered at the country level reported in parentheses, with significance levels as follows: *** p<0.01, ** p<0.05, * p<0.10. Regressions are weighted to be representative of the < 5 year population of children of all countries included in this DHS dataset through a three-step weighting procedure factoring in country <5 year population size, DHS round sample size and conventional DHS survey weights. The coefficients in these regressions can also be interpreted as elasticities as follows: a. This elasticity is the coefficient on the interaction between annual change in GNI per capita lagged one year and the country-specific prevalence of wasting averaged over all DHS rounds, defined for each specific wasting indicator. The coefficient therefore represents the elasticity of wasting b. This is analogous coefficient/elasticity for lagged growth in GDP per capita. The regressions control for various factors not reported for the sake of brevity: c. DHS child, maternal and household effects include household asset ownership, maternal education years, piped water and flush toilet access, whether the child was born in a medical facility, whether the mother received four or more antenatal care visits, whether the child received all vaccinations, whether the child was born of a teenage pregnancy, whether the mother has four or more children, whether the child is female, and resides in a rural area. d. Temporal effects include region-specific seasonality effects using month of survey dummies, wasting-age dynamics captured by child age dummies, and time trend effects captured by 5-year time dummies. GDP=Gross Domestic Product and GNI=Gross National Income. WHZ=Weight for height Z score.

Supplementary Table 5: Multivariate linear probability models of wasting risks as a function of lagged GDP growth shocks, with interactions for urban location and female status (children 0-59 months)

	Any wasting (WHZ < -1)	Moderate/severe wasting (WHZ < -2)	Severe wasting (WHZ < -3)
	(1) N=1,256,076	(2) N=1,256,076	(3) N=1,256,076
GDP growth elasticity ($w.g^n$)	-0.0679*** (-0.152, -0.005)	-0.167** (-0.296, -0.038)	-0.291*** (-0.514, -0.067)
GDP growth elasticity ($w.g^n$)*urban dummy	-0.047* (-0.096, 0.004)	-0.053** (-0.099, -0.006)	0.004 (-0.126, 0.121)
R-squared	0.432	0.200	0.085
	(4) N=1,256,076	(5) N=1,256,076	(6) N=1,256,076
GDP growth elasticity ($w.g^n$)	-0.096*** (-0.171, -0.021)	-0.163*** (-0.299, -0.028)	-0.26*** (-0.513, -0.011)
GDP growth elasticity ($w.g^n$)*girl dummy	0.016 (-0.004, 0.037)	-0.026* (-0.077, 0.026)	-0.050** (-0.121, 0.003)
R-squared	0.432	0.200	0.085
DHS child, mother, household effects?[c]	Yes	Yes	Yes
Country fixed effects?	Yes	Yes	Yes
Region-specific temporal effects?[d]	Yes	Yes	Yes

Notes: *N* = 1,256,076 children in all regressions. Results are linear probability model coefficient point estimates with 95% confidence intervals based on standard errors clustered at the country level reported in parentheses, with significance levels as follows: *** p<0.01, ** p<0.05, * p<0.10. Regressions are weighted to be representative of the < 5 year population of children of all countries included in this DHS dataset through a three-step weighting procedure factoring in country <5 year population size, DHS round sample size and conventional DHS survey weights. The coefficients in these regressions can also be interpreted as elasticities as follows: a. This elasticity is the coefficient on the interaction between annual change in GDP per capita lagged one year and the country-specific prevalence of wasting averaged over all DHS rounds, defined for each specific wasting indicator. The coefficient therefore represents the elasticity of wasting b. This is analogous coefficient/elasticity for lagged growth in GDP per capita. The regressions control for various factors not reported for the sake of brevity: c. DHS child, maternal and household effects include household asset ownership, maternal education years, piped water and flush toilet access, whether the child was born in a medical facility, whether the mother received four or more antenatal care visits, whether the child received all vaccinations, whether the child was born of a teenage pregnancy, whether the mother has four or more children, whether the child is female, and resides in a rural area. d. Temporal effects include region-specific seasonality effects using month of survey dummies, wasting-age dynamics captured by child age dummies, and time trend effects captured by 5-year time dummies.
GDP=Gross Domestic Product and GNI=Gross National Income. WHZ=Weight for height Z score.

Supplementary Fig. 5: Elasticities of children's consumption of various foods with respect to GNI growth or GDP growth (90% CIs)

Panel A: GNI growth

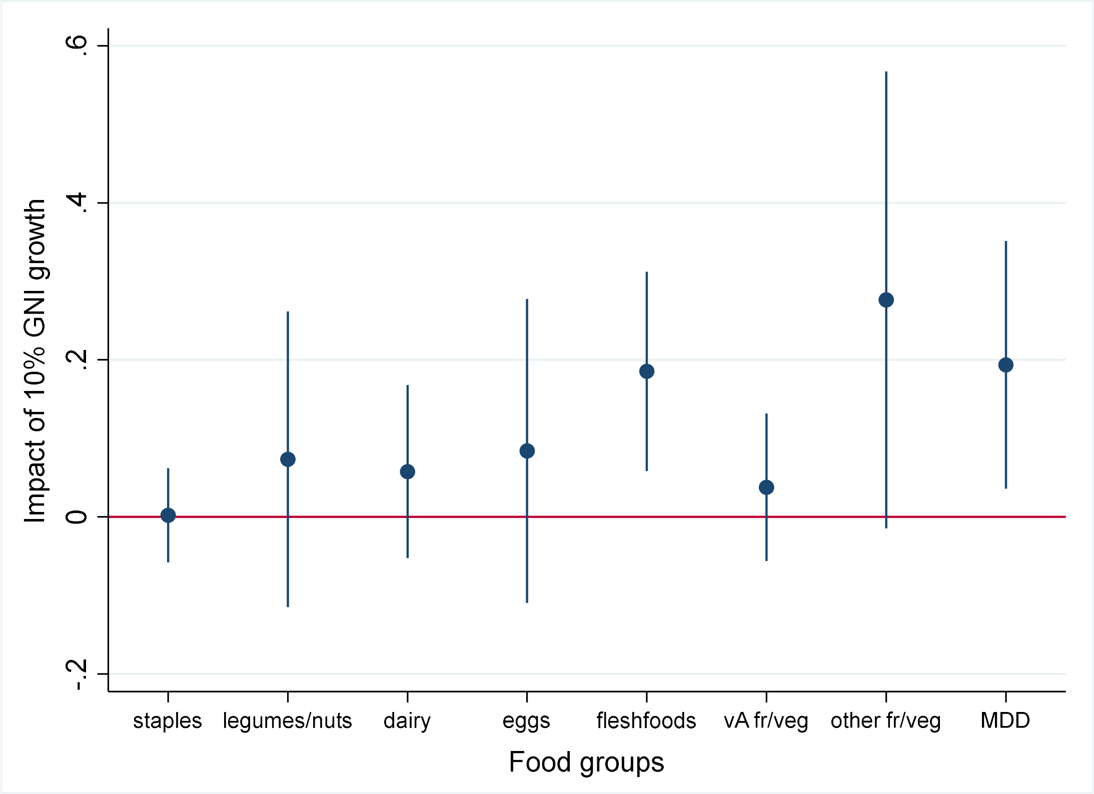

Panel B: GDP growth

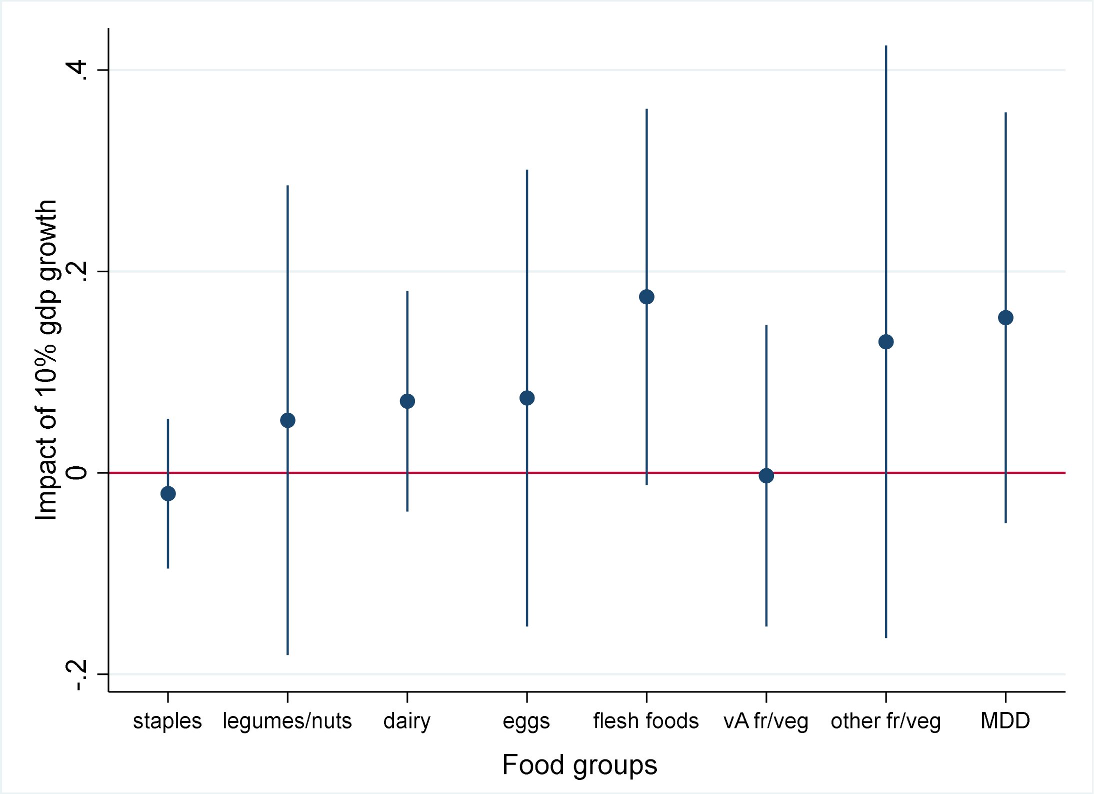

N = 390,361 children in all regressions. Results are linear probability model coefficient point estimates of the impact of GNI or GDP shocks on whether a child consumed each food group in the past 24 hours, with 95% confidence intervals based on standard errors clustered at the country level reported in parentheses, with significance levels as follows: *** p<0.01, ** p<0.05, * p<0.10. . "staples" includes grains, roots, tubers. "flesh foods" refers to meat, organs and fish. "vA fr/veg" refers to vitamin A-rich fruits and vegetables, while "other fr/veg" refers to all other fruits and vegetables. MDD refers to minimum dietary diversity. Regressions are weighted to be representative of the < 5 year population of children of all countries included in this DHS dataset through a three-step weighting procedure factoring in country <5 year population size, DHS round sample size and conventional DHS survey weights. The regressions control for various factors not reported for the sake of brevity: DHS child, maternal and household effects include household asset ownership, maternal education years, piped water and flush toilet access, whether the child was born in a medical facility, whether the mother received four or more antenatal care visits, whether the child received all vaccinations, whether the child was born of a teenage pregnancy, whether the mother has four or more children, whether the child is female, and resides in a rural area. Temporal effects include region-specific seasonality effects using month of survey dummies, wasting-age dynamics captured by child age dummies, and time trend effects captured by 5-year time dummies. GDP=Gross Domestic Product and GNI=Gross National Income.

Supplementary Table 6: Associations between moderate/severe wasting and morbidity symptoms, maternal underweight and child minimum dietary diversity

	(1)	(2)
	Full sample	Child diets sample
Dependent variable	Moderate or severe wasting (WHZ< -2)	Moderate or severe wasting (WHZ< -2)
Age range	0-59m	6-35m
Diarrhea in past 2 weeks	0.018***	0.018***
	(0.014, 0.022)	(0.015, 0.020)
Fever-only in past 2 weeks	0.016***	0.025***
	(0.009, 0.023)	(0.016, 0.035)
Low maternal BMI	0.068***	0.075***
	(0.061, 0.075)	(0.071, 0.080)
Minimum diet diversity		-0.015***
		(-0.022, -0.008)
R-squared	0.072	0.073
DHS child, mother, household effects?[c]	Yes	Yes
Country fixed effects?	Yes	Yes
Region-specific temporal effects?[d]	Yes	Yes

Notes: *N* = 1,174,216 for the Full sample in regression (1) and *N* = 302,542 for the Child diets sample in regression (*2).* Results in regression (1) are linear probability model coefficient point estimates of the association between diarrhea and fever-only in the past 2 weeks and Low maternal BMI on moderate or severe wasting, with 95% confidence intervals based on standard errors clustered at the country level reported in parentheses, with significance levels as follows:: *** p<0.01, ** p<0.05, * p<0.10. All regressions control for country fixed effects, region-specific seasonality effects, wasting-age dynamics and trend effects. Regressions are weighted to be representative of the < 5 years population of children of all countries included in this DHS dataset. WHZ=Weight for height Z score.
