# Supplementary material for: Economic shocks predict increases in child wasting prevalence
Source: Nat Commun. 2022 Apr 20;13:2157. doi: 10.1038/s41467-022-29755-x (PMC9021262; doi:10.1038/s41467-022-29755-x)
Supplement: Supplementary file 3 — Reporting Summary [file 41467_2022_29755_MOESM3_ESM.pdf]

nature research

Corresponding author(s): NCOMMS-20-22867A (Derek Headey)

Last updated by author(s): Mar 1, 2022

Reporting Summary

Nature Research wishes to improve the reproducibility of the work that we publish. This form provides structure for consistency and transparency in reporting. For further information on Nature Research policies, see our Editorial Policies and the Editorial Policy Checklist.

Statistics

For all statistical analyses, confirm that the following items are present in the figure legend, table legend, main text, or Methods section.

n/a	Confirmed	
☐	☒	The exact sample size (*n*) for each experimental group/condition, given as a discrete number and unit of measurement
☒	☐	A statement on whether measurements were taken from distinct samples or whether the same sample was measured repeatedly
☐	☒	The statistical test(s) used AND whether they are one- or two-sided<br>*Only common tests should be described solely by name; describe more complex techniques in the Methods section.*
☐	☒	A description of all covariates tested
☐	☒	A description of any assumptions or corrections, such as tests of normality and adjustment for multiple comparisons
☐	☒	A full description of the statistical parameters including central tendency (e.g. means) or other basic estimates (e.g. regression coefficient) AND variation (e.g. standard deviation) or associated estimates of uncertainty (e.g. confidence intervals)
☐	☒	For null hypothesis testing, the test statistic (e.g. *F*, *t*, *r*) with confidence intervals, effect sizes, degrees of freedom and *P* value noted<br>*Give P values as exact values whenever suitable.*
☒	☐	For Bayesian analysis, information on the choice of priors and Markov chain Monte Carlo settings
☒	☐	For hierarchical and complex designs, identification of the appropriate level for tests and full reporting of outcomes
☒	☐	Estimates of effect sizes (e.g. Cohen's *d*, Pearson's *r*), indicating how they were calculated

Our web collection on statistics for biologists contains articles on many of the points above.

Software and code

Policy information about availability of computer code

Data collection	STATA Version 16 was used to merge together Demographic Health Survey data with UN and World Bank national accounts data and other national level covariates.
Data analysis	STATA Version 16 was used to calculate descriptive statistics and implemented non-parametric and parametric regression analysis. The STATA code for the analysis has been made available on Zenodo, with DOI: https://doi.org/10.5281/zenodo.6318072.

For manuscripts utilizing custom algorithms or software that are central to the research but not yet described in published literature, software must be made available to editors and reviewers. We strongly encourage code deposition in a community repository (e.g. GitHub). See the Nature Research guidelines for submitting code & software for further information.

Data

Policy information about availability of data

All manuscripts must include a data availability statement. This statement should provide the following information, where applicable:

- Accession codes, unique identifiers, or web links for publicly available datasets
- A list of figures that have associated raw data
- A description of any restrictions on data availability

This analysis uses three sources of data which were merged into a single dataset. The main dataset is a child level dataset compiling 177 national level Demographic Health Surveys from 52 countries implemented over 1990-2018. These data were downloaded from https://dhsprogram.com/data/available-datasets.cfm and are freely available after registration on the DHS website. The individual survey rounds are listed in Appendix Table A1 in our paper. These 177 surveys were then stacked and variables from UN and World Bank sources were then merged to the year of interview or its 1-year lag. UN National Accounts data is freely accessible here: https://unstats.un.org/unsd/snaama/. World Bank national accounts data and information on battle deaths is freely available here: https://databank.worldbank.org/source/world-development-indicators. Data availability: The data that support the findings of this study are available from The

Demographic Health Surveys (https://dhsprogram.com/data/available-datasets.cfm) but restrictions apply to the availability of these data, which were used under license for the current study, and so are not publicly available. Data are however available from the authors upon reasonable request and with permission of the DHS. UN and World Bank macroeconomic indicators are publicly available. The authors are committed to sharing their data with any interested researchers.

Field-specific reporting

Please select the one below that is the best fit for your research. If you are not sure, read the appropriate sections before making your selection.

☐ Life sciences ☒ Behavioural & social sciences ☐ Ecological, evolutionary & environmental sciences

For a reference copy of the document with all sections, see nature.com/documents/nr-reporting-summary-flat.pdf

Behavioural & social sciences study design

All studies must disclose on these points even when the disclosure is negative.

Study description

This is a quantitative study linking together secondary datasets - micro data from household surveys with macroeconomic indicators from national accounts databases - to test whether national income growth rates affect changes in wasting risks (low weight-for-height) among children 0-59 months of age.

Research sample

We use a sample of children 0-59 months of age surveyed in 177 nationally representative Demographic Health Surveys (DHS) implemented in 52 low and middle income countries between 1990-2018. Our primary outcome is a child's wasting status, defined as whether their weight-for-height z scores (relative to international reference standards) are significantly below various thresholds (WHZ<-1, <-2, <-3). Wasting status is then linked to national income growth rates in the year prior to each child's recorded measurement/survey. The sample was chosen based on the fact that wasting prevalence is highest in the first few years of life, typically peaking around age 12 months as shown in this study (FIgure 1) and in Headey and Alderman: lderman, H. & Headey, D. The timing of growth faltering has important implications for observational analyses of the underlying determinants of nutrition outcomes. PLOS ONE 13, e0195904, doi:10.1371/journal.pone.0195904 (2018).

Sampling strategy

Demographic Health Surveys collection nationally representative samples through a complex two-stage stratified sampling design, with household weights used to derive nationally and subnationally representative sampling statistics. We used all available Demographic Health Surveys as of January 2020, provided they collected anthropometric measurements for all children 0-59 months of age. The DHS are widely used and their sampling approach is described in detail online: https://dhsprogram.com/Data/Guide-to-DHS-Statistics/index.cfm

Data collection

We used secondary datasets as described and collected all available Demographic Health Surveys as of January 2020. The basic approach of The DHS Program is to collect data that are comparable across countries. To achieve this, standard model questionnaires have been developed, along with a written description of why certain questions or sections have been included. These model questionnaires—which have been reviewed and modified in each of the six phases of The DHS Program—form the basis for the questionnaires that are applied in each country. Typically, a country is asked to adopt the model questionnaire in its entirety, but can add questions of particular interest. However, questions in the model can be deleted if they are irrelevant in a particular country. The survey was multi-purpose and not designed for experimental purposes, hence participants were not blinded to the objectives of the survey, and were instead informed about the survey objectives and likely uses.
See https://dhsprogram.com/Data/Guide-to-DHS-Statistics/index.cfm

Timing

Demographic Health Surveys with the full range of anthropometric data for children 0-59 months of age were first collected in 1990 and most recently collected in 2018. The number of surveys conducted within each country varies, with some countries collecting data at higher frequency (1-3 years) and others less frequently (5-10 years). The surveys used in this analysis are described in Supplementary Table S1 and S2.

Data exclusions

Demographic Health Surveys that did not collect anthropometric data for all children 0-59 months were excluded, as well as countries in which only one survey round was conducted, thereby precluding the use of country fixed effects. This resulted in 24 surveys being excluded.

Non-participation

This is not applicable to this study as it used secondary datasets.

Randomization

The DHS uses randomized sampling with a two-stage stratified sampling design.

Reporting for specific materials, systems and methods

We require information from authors about some types of materials, experimental systems and methods used in many studies. Here, indicate whether each material, system or method listed is relevant to your study. If you are not sure if a list item applies to your research, read the appropriate section before selecting a response.

Materials & experimental systems

n/a	Involved in the study
☒	☐ Antibodies
☒	☐ Eukaryotic cell lines
☒	☐ Palaeontology and archaeology
☒	☐ Animals and other organisms
☒	☐ Human research participants
☒	☐ Clinical data
☒	☐ Dual use research of concern

Methods

n/a	Involved in the study
☒	☐ ChIP-seq
☒	☐ Flow cytometry
☒	☐ MRI-based neuroimaging
